# Protease Inhibitory Effect of Natural Polyphenolic Compounds on SARS-CoV-2: An In Silico Study

**DOI:** 10.3390/molecules25204604

**Published:** 2020-10-10

**Authors:** Rajveer Singh, Anupam Gautam, Shivani Chandel, Arijit Ghosh, Dhritiman Dey, Syamal Roy, Velayutham Ravichandiran, Dipanjan Ghosh

**Affiliations:** 1 National Institute of Pharmaceutical Education and Research, Kolkata 70054, India; rajveersidhu94@gmail.com (R.S.); chandelshivani48@gmail.com (S.C.); dhritimandey@gmail.com (D.D.); drsyamalroy@yahoo.com (S.R.); directorniperkolkata@gmail.com (V.R.); 2 Institute for Bioinformatics and Medical Informatics, University of Tübingen, Sand 14, 72076 Tübingen, Germany; anupam.gautam@uni-tuebingen.de; 3 International Max Planck Research School ‘From Molecules to Organisms’, 72076 Tübingen, Germany; 4 Department of Chemistry, University of Calcutta, Kolkata 700009, India; jitbiochem71@gmail.com

**Keywords:** polyphenols, SARS-CoV-2, molecular docking, molecular dynamic simulation, MM-PBSA

## Abstract

The current pandemic, caused by SARS-CoV-2 virus, is a severe challenge for human health and the world economy. There is an urgent need for development of drugs that can manage this pandemic, as it has already infected 19 million people and led to the death of around 711,277 people worldwide. At this time, in-silico studies are providing lots of preliminary data about potential drugs, which can be a great help in further in-vitro and in-vivo studies. Here, we have selected three polyphenolic compounds, mangiferin, glucogallin, and phlorizin. These compounds are isolated from different natural sources but share structural similarities and have been reported for their antiviral activity. The objective of this study is to analyze and predict the anti-protease activity of these compounds on SARS-CoV-2main protease (Mpro) and TMPRSS2 protein. Both the viral protein and the host protein play an important role in the viral life cycle, such as post-translational modification and viral spike protein priming. This study has been performed by molecular docking of the compounds using PyRx with AutoDock Vina on the two aforementioned targets chosen for this study, i.e., SARS-CoV-2 Mpro and TMPRSS2. The compounds showed good binding affinity and are further analyzed by (Molecular dynamic) MD and Molecular Mechanics Poisson-Boltzmann Surface Area MM-PBSA study. The MD-simulation study has predicted that these natural compounds will have a great impact on the stabilization of the binding cavity of the Mpro of SARS-CoV-2. The predicted pharmacokinetic parameters also show that these compounds are expected to have good solubility and absorption properties. Further predictions for these compounds also showed no involvement in drug-drug interaction and no toxicity.

## 1. Introduction

The current COVID-19 pandemic is the most serious global outbreak to have occurred in recent times. Researchers from different fields are trying to understand it by different approaches [1]. However, until now, no medication or any established therapy has been established. Natural compounds always offer a great promise in the cure of critical diseases. Polyphenolic compounds are naturally occurring phenolic compounds that contain more than one phenol group. Polyphenols are found largely in the fruits, vegetables, cereals. It has been observed that fruits such as apples, pears, grapes, and cherries contain as much as 200 to 300 mg of polyphenolic compounds per 100 g of weight. Phenolic acids, flavonoids, and tannins are found to be the main dietary compounds among several classes of phenolic compounds [2]. Dietary intake of polyphenol-rich foods in high amounts has been confirmed to reduce the incidence of several diseases such as cardiovascular diseases, diabetes mellitus, osteoporosis, neurodegenerative diseases, and cancer. For a long time, in-vitro and in-vivo studies has been showing the potentiality of polyphenolic compounds as therapeutic agents such as cardio-protective [3], anti-diabetic [4], anti-cancer [5], neuroprotective [6], and antimicrobial agents [7,8]. Considering these medicinal potentialities of polyphenolic compounds, we have selected three polyphenolic compounds for our study that have structural similarity, i.e., mangiferin, glucogallin, and phlorizin which are present in fruits such as mango, amla, and apple, respectively.

The novel coronavirus is deadly due to its high binding affinity on angiotensin-converting enzyme 2 (ACE-2) in the alveoli of the lungs [9]. Now it is known that SARS-2-S uses angiotensin-converting enzyme II (ACE-2) as the entry receptor to enter into the host cell and uses the host serine protease TMPRSS2 for S protein priming, so that it can bind with the ACE-2 [9]. Whereas, for viral assembly, trafficking, and release of viral-like particles, M proteins are important. TMPRSS2 has been used by not only SARS-CoV-2 but also other types of coronaviruses and influenza viruses for viral S protein priming and cell entry, including SARS-CoV as well as influenza H1N1 [10,11,12]. Just like SARS-CoV-I, the SARS-CoV-2 spike protein firstly attaches to (ACE-2), encoded by the ACE-2 gene, which is expressed on respiratory epithelial cells. In a second step, the spike protein is being cleaved by the proteases present on the host cell, particularly TMPRSS2, to activate the viral entry [10].

The mangiferin is the main important constituent of mangifera indica and was reported to possess antiviral activity against the herpes simplex virus 1 and 2 (HSV1 and HSV-2) [13]. Another important activity of mangiferin is the immuno-stimulatory activity, and it also has a protective effect on the respiratory system [13,14,15,16]. The phlorizin is the glucoside derivative of phloretin, which is found in the unripe apple and bark of the apple. This compound has structural similarity with the mangiferin, both having the glucoside moiety. Phlorizin has been reported to have a synergistic effect with antiviral drugs such as ritonavir with an antioxidant and also has cardio-protectant properties [17]. Glucogallin, belonging to the class of gallotannin, is biosynthesized from gallic acid and UDP-glucose. It is mainly found in the oak leave and amla fruit and has strong antiviral properties against the simplex virus 1 [18].

We have analyzed the potential of the three polyphenols, i.e., mangiferin, glucogallin, and phlorizin as protease inhibitors against the crystal structure of SARS-CoV-2 main protease (PDB ID-6LU7) and TMPRSS2 homology model by molecular docking and molecular dynamics study. We have also done the absorption, distribution, metabolism and excretion ADME analysis, toxicity prediction, and target prediction of these polyphenolic compounds.

## 2. Results

### 2.1. Homology Modelling

**Figure 1:**
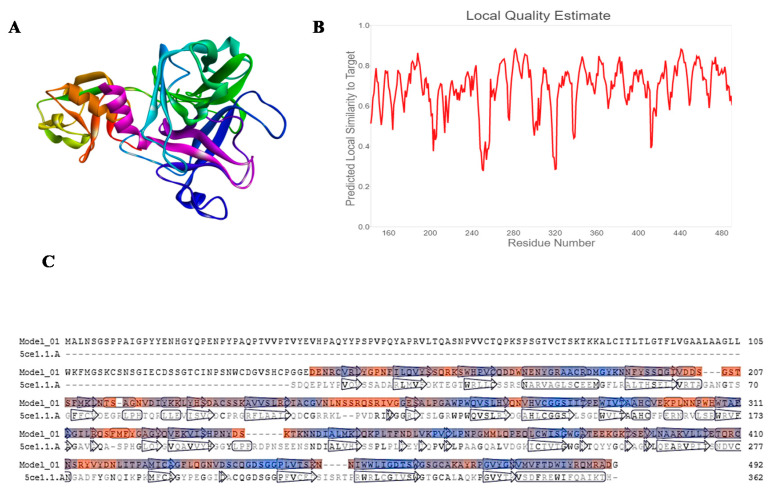
(**A**) Homology model of TMPRSS2, (**B**) predicted local quality estimation with chart of target by SWISS-Model server, and (**C**) sequence alignment between model target(TMPRSS2) and Human Hepsin TMPRSS1 (Human Hepsin TMPRSS1 (5ce1.1.A).1.A.

SWISS-MODEL server has developed three different models of TMPRSS2, out of which we have selected the best model for molecular docking based upon the QMEANS score. The homology model is considered as good and reliable if the target sequence was found to be more than 30%. Sequence alignment for the TMPRSS2 and Human Hepsin TMPRSS1 (5ce1.1.A) shows a similarity of 33.82% (Figure 1). The models were built depending on the template-target alignment utilizing the ProMod3. The geometry of the model was regularized by utilizing the force field. The quality was assessed by the QMEAN scoring function. QMEANS value [19,20] and Generalized Quantum Master Equations (GMQE) value of the model was found to be −1.43 and 0.53, respectively. We further analyzed the homology model of TMPRSS2 (344 amino acid residues) with the help of RAMPAGE software and noticed that 92.7% of the total residues were in favored regions, 6.7% of the total residues were in allowed regions and only 0.6% of the total residues were in outlier region (Supplementary Material Figure S1).

### 2.2. Molecular Docking

We have selected three natural compounds from different natural source for molecular docking against the main proteases of SARS-CoV-2 (Mpro) and TMPRSS2. These compounds have previous reports of anti-viral properties and structural similarities. The binding affinities of the compounds are listed in the tables for the Mpro of SARS-CoV-2 and TMPRSS2 protein.

**Figure 2:**
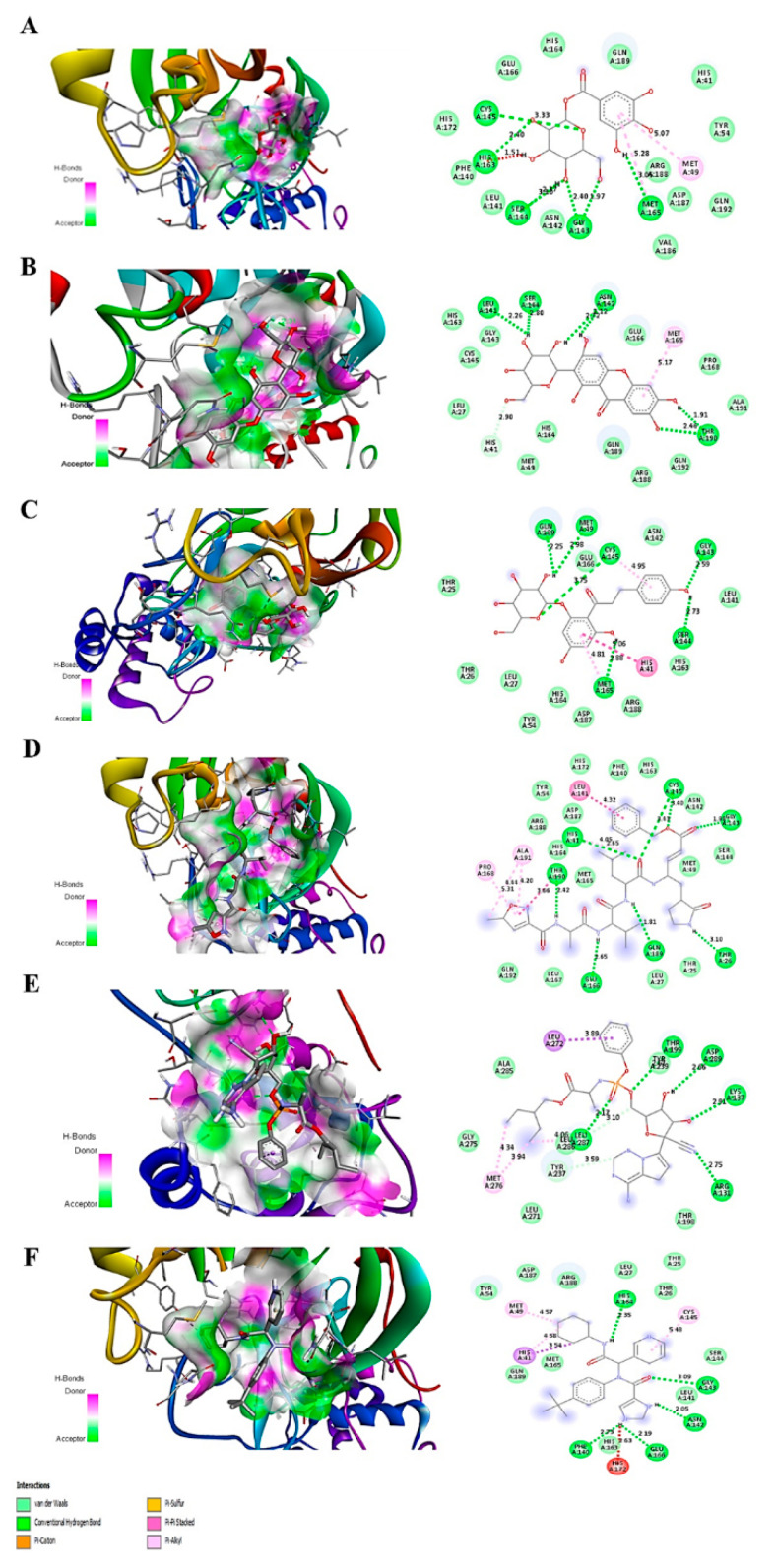
Predicted 3D structure of the ligand-protein interaction at the left side and 2D animated pose showing interaction. (**A**) Main proteases protein of SARS-CoV-2 with glucogallin. (**B**) Main proteases protein of SARS-CoV-2 with mangiferin. (**C**) Main proteases protein of SARS-CoV-2 with phlorizin. (**D**) Main proteases protein of SARS-CoV-2 with N3. (**E**) Main proteases protein of SARS-CoV-2 with remdesivir. (**F**) Main proteases protein of SARS-CoV-2 with X77.

Mangiferin showed the highest binding affinity for Mpro, which is −8.5kcal/mol, while the binding affinity of glucogallin and phlorizin was −7 kcal/mol −7.9 kcal/mol, respectively. The binding and mode of interactions of these compounds with Mpro are shown in Figure 2. From the interactions, it has been shown that primarily there are three types of interaction (H bonding, electrostatic, van der Waals forces). Mangiferin showed conventional H bond interaction with HIS-164(A), SER144(A), HIS41(A), ASN142(A), THR190(A); van der Waals interaction with ALA191(A), GLN192(A), ARG188(A), LEU167(A), PRO168(A), GLN189(A), GLU166(A), LEU141(A), HIS163(A), CYS145(A), GLY143(A), MET49(A), and hydrophobic interaction with MET165(A) at the active site of main proteases. Whereas, glucogallin showed conventional hydrogen bond with CYS145 (A), SER144 (A), GLY143 (A), MET165 (A); van der Waals interaction with PHE140(A), HIS164(A), GLU166(A), GLN189(A), ARG188(A), and hydrophobic interaction with MET49(A). Similarly, the third compound, phlorizin, showed conventional H bonds with GLN189(A), MET49(A), CYS145(A), MET165(A), GLY143(A), and SER144(A);van der Waals interaction with THR25(A), THR26(A), LEU27(A), TYR54(A), ASP187(A), HIS164(A), ARG188(A), HIS163(A), LEU141(A), ASN142(A). We have also compared the binding affinity of our compounds with three different reference compounds remdesivir, X77, and N3 for Mpro with previously reported docking scores of −7.2 kcal/mol, −8.23 kcal/mol, and −7.9 kcal/mol, respectively, which is near to our obtained docking scores of −7.9 kcal/mol, −7.5 kcal/mol, and−8.6 kcal/mol [21,22,23]. Remdesivir showed five conventional H bonds with THR199(A), ASP289(A), LYS137(A), ARG 131(A), LEU287(A); van der Waals interaction with ALA285(A), GLY275(A), LEU271(A), ASN238(A), THR198(A), ASP197(A), TYR239(A). The N3 showed conventional H bonds with HIS41(A), THR190(A), GLU166(A), GLN189(A), THR26(A), GLY143(A), CYS145(A); van der Waals interaction with GLN192(A), LEU167(A), LEU27(A), THR25(A), MET49(A), SER144(A), ASN142(A), HIS163(A), PHE140(A), HIS172(A), TYR54(A) ARG 188(A), ASP187, HIS164, MET165. The X77 compound showed 5 hydrogen bonds with HIS164(A), GLY143(A), ASN142(A), GLU166(A), PHE140(A); van der Waals interaction with GLN189(A), MET165(A), TYR54(A), ASP187(A), ARG188(A), LEU 27(A), THR25(A), THR 26(A), SER144(A), LEU141(A), HIS163(A). The predicted interacting residues are summarized in (Table 1).

**Figure 3:**
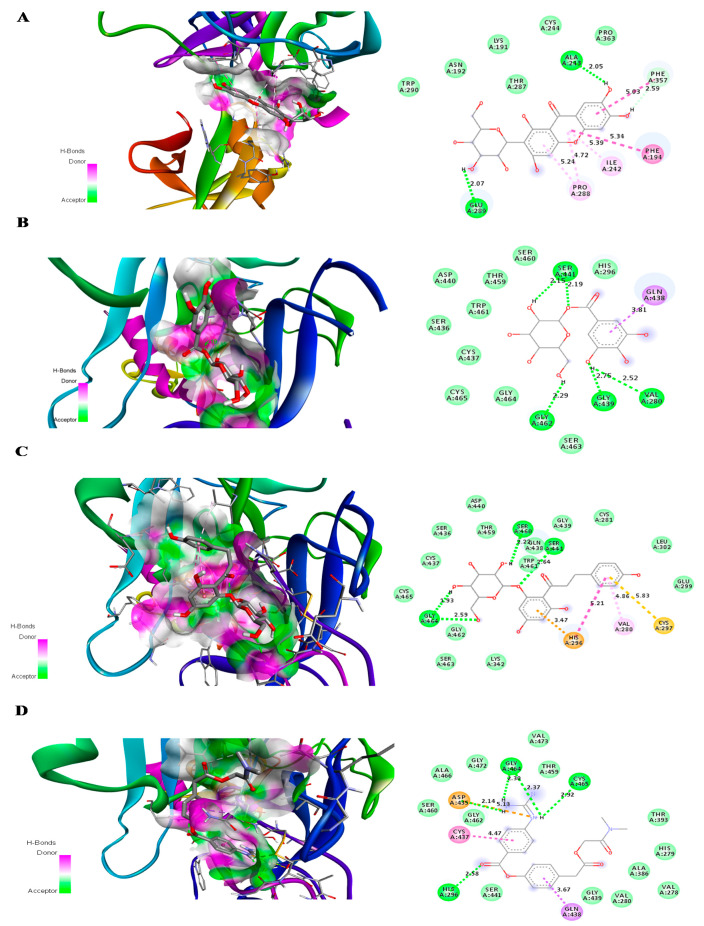
Predicted 3D structure of the ligand–protein interaction at the left side and 2D animated pose showing interaction. (**A**) TMPRSS2 protein of SARS-CoV-2 with mangiferin. (**B**) TMPRSS2 protein of SARS-CoV-2 with glucogallin. (**C**) TMPRSS2 protein of SARS-CoV-2 with phlorizin. (**D**) TMPRSS2 protein of SARS-CoV-2 with camostat mesylate.

For TMPRSS2 protein, phlorizin showed the highest binding affinity with a binding energy of −7.7 kcal/mol. The binding energy of both glucogallin and mangiferin was found to be −6.9 kcal/mol. The binding residues and binding modes of these compounds are shown in Figure 3. Mangiferin showed the conventional H bonding interaction with residues ALA243 (A) and GLU289 (A). Van der Waals interaction of mangiferin was found to be with the residues TRP290 (A), ASN192 (A), LYS191 (A), CYS244 (A), THR287 (A), and PRO363 (A) of TMPRSS2 mangiferin showed pi-donor hydrogen binding interaction with residues PHE 357 (A), Pi-Pi alkyl interaction with ILE242 (A), and PRO288 (A). Glucogallin showed conventional H-bonds with residues SER441 (A), VAL280 (A), GLY439 (A), and GLY462 (A); van der Waals interaction with residues HIS296 (A), SER460 (A), THR459 (A), ASP440 (A), TRP461 (A), SER436 (A), CYS437 (A), CYS464 (A), SER463 (A), and Pi-Sigma bonding with GLN438 (A). Phlorizin showed the highest binding with TMPRSS2 protein, showing conventional hydrogen bonds with residues SER460 (A), SER441 (A), and GLY464 (A). The van der Waals interaction between phlorizin and TMPRSS2 was found with residues CYS465 (A), CYS437 (A), SER436 (A), ASP440 (A), THR459 (A), GLN438 (A), TRP461 (A), GLY462 (A), SER463 (A), LYS342 (A), GLY439 (A), CYS281 (A), LEU302 (A), and GLU299 (A). The phlorizin showed pi-cation, pi-alkyl, and pi-sulfur bonding with the residues HIS-296 (A), VAL280 (A), and CYS297 (A), respectively. We also analyzed the binding affinity of camostat mesylate for the TMPRSS2. It showed the conventional hydrogen bonds with residues GLY464(A), CYS465(A), HIS296(A),and ASP 435(A); van der Waals interaction with residues ALA466(A), SER460(A), GLY462(A), ALA466(A), GLY472(A), TRP461(A), VAL473(A), THR459(A), THR393(A), HIS279(A), ALA386(A), VAL278(A), VAL280(A), GLY439(A), and SER441 (A) (Table 2). We have used DockThor server [26] to cross check the results of our docking study. The docking score from this server showed docking scores near to PyRx AutoDock Vina (Tables S5 and S6). After analyzing the docking scores of the reference compounds obtained from both of the servers with the docking scores that are already published, we observed that the published docking scores of remdesivir and N3 are near to the docking scores that are obtained from the PyRx AutoDock Vina software. Whereas, the published docking score of X77 are near to the docking score obtained from the DockThor server.

### 2.3. ADME Prediction

The absorption, distribution, metabolism, and excretion (ADME) parameters have been analyzed by the pkCSM database, which gives the result after submission of small molecules in smiles format or simply providing the linear structure. The molecular properties of the compounds have been mentioned in the supplementary data. The pharmacokinetic parameter of the three compounds showed less absorption from the GIT tract, no BBB permeability, and no effect on the CYP2D6, CYP3A4, CYP1A2, CYP2C19, CYP2C9, CYP2D6, CYP3A4 cytochrome enzymes (Tables S1–S4).

### 2.4. Prediction of Targets

**Figure 4:**
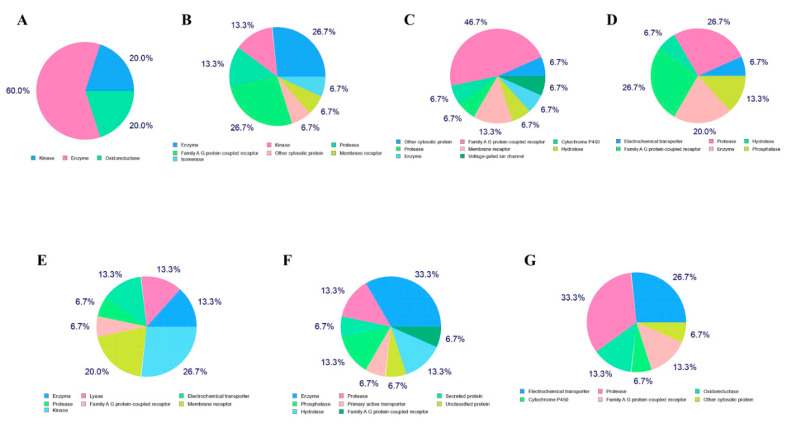
Target prediction, (**A**) remdesivir, (**B**) X77, (**C**) N3, (**D**) camostat mesylate, (**E**) mangiferin, (**F**) glucogallin, and (**G**) phlorizin.

The prediction of targets was analyzed by the online Swiss Target Prediction server, which gives the top 15 results in the form of pie-chart (Figure 4). In the case of mangiferin the pie chart showed probable protein target type as 26.7% enzyme, 13.3 kinases, 6.7% proteases, 13.3% lyase, 6.7% family A G-protein-coupled receptor, 20% membrane receptor. The pie chart of glucogallin showed 33.3% enzyme, 13.3% phosphatases, 13.3% hydrolases, 13.3% proteases, 6.7% primary active transporter, and family A G protein-coupled receptor. The pie chart of phlorizin showed that 33.3% proteases, 26.7% of electrochemical transporter, 13.3% cytochrome P450, 13.3% of family A G-protein-coupled receptor, 6.7% of oxidoreductase, and other cytosolic protein (Figure 4). These were the possible target sites to which the compounds may bind which were predicted by the server.

### 2.5. Prediction of Toxicity

The toxicity study along with the reference compounds was performed with the pkCSM database, which predicted that the selected natural compounds do not have any AMES toxicity, hepatotoxicity, and skin sensitivity. Whereas all the reference compounds may possess hepatotoxicity, and X77 and N3 may possess AMES toxicity as predicted by the server. The natural compounds also may not have the hERGI and hERGII inhibition activity, but all the reference compounds may have hERGII inhibition activity (Table 3).

### 2.6. Molecular Dynamics Simulation and MM-PBSA Analysis

Molecular dynamic simulation (MDS) offers a great insight into the proper binding of ligand candidates with the protein under analysis. A workflow combined with molecular docking, molecular dynamic, and free energy calculation has been engaged in understanding the properties of individual natural products in solvation state. In this study, a 10 ns MDS was performed to calculate the binding affinity and conformational stability of natural products to the Mpro and TMPRSS2. Reference compounds such as remdesivir, N3, and X77 for Mpro and camostat mesylate for TMPRSS2 were selected along with the polyphenols, mangiferin, glucogallin, and phlorizin for the MDS study. The MDS trajectories were screened out on the following parameters—Root Mean Square Deviation (RMSD), Root Mean Square Fluctuation (RMSF), Solvent-accessible surface area (SASA), Rg, and binding free energies. RMSD plot of phlorizin-Mpro complex achieves the conformational stability at 2.5 ns with RMSD of 0.2 nm and stayed at the same conformation up to 7.5 ns. The glucogallin-Mpro protein complex achieved optimum confirmation of 0.1 nm at around 1.25 ns and remained unchanged until 10 ns. RMSD plot of the mangiferin-Mpro showed very stable conformation around 0.1 nm, starting from 1 ns to 10 ns. The RMSD plots of all the three ligand-protein complexes showed very stable conformation throughout the simulation study, which demonstrates that it has a huge impact on the Mpro target (Figure S2A) as the reference compound N3. In case of TMPRSS2, the phlorizin-TMPRSS2 complex became stable after 2.5 ns and stays stable up to 10 ns with RMSD of less than 0.3 nm. The RMSD plot of glucogallin-TMPRSS2 complex started above 0.2 nm and remained stable from 2 ns to 10 ns. The mangiferin-TMPRSS2 complex achieved the conformation stability after 2.5 ns having the RMSD value more than 0.2 nm. All the ligand-TMPRSS2 complexes having low RMSD value with little fluctuation showed that the ligands have a huge impact on the protein structures (Figure S2B).

**Figure 5:**
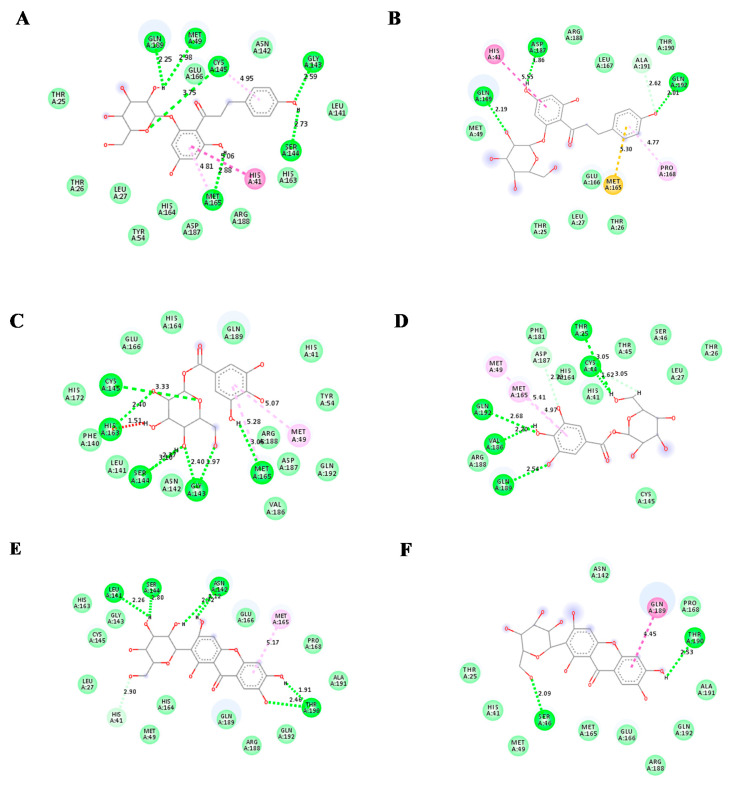
(**A**) Docked phlorizin-Mpro complex (t = 0), (**B**) phlorizin-Mpro complex after MD (t = 10ns), (**C**) Docked glucogallin-Mpro complex (t = 0), (**D**) glucogallin-Mpro complex after MD (t = 10ns), (**E**) Docked mangiferin-Mpro complex (t = 0), (**F**) mangiferin-Mpro complex after MD (t = 10ns).

Further analysis of the protein-ligand interaction of the protein ligand complexes after the simulation process, it revealed that for the phlorizin-Mpro complex, three new conventional hydrogen bonds with residues GLN 189 (A), ASP 187 (A), and GLN 192 (A) have been formed. This stabilizes the complex during the MDS process (Figure 5B). The analysis of binding mode of the glucogallin with receptor binding domain (RBD) of main protease (Mpro) after MDS showed formation of five new conventional hydrogen bonds with GLN 192 (A), VAL186 (A), GLN 189 (A), THR 25 (A), and CYS 44 (A) that has been elaborated in Figure 5C,D. The newly made conventional hydrogen bonds with shorter bond length lowered down the RMSD value of glucogallin-Mpro complex (Figure 5D). To shed light on the third complex, i.e., mangiferin-Mpro complex, after MDS only one new hydrogen bond has been formed at SER 46 (A), which lowers the RMSD value (Figure 5F).

**Figure 6:**
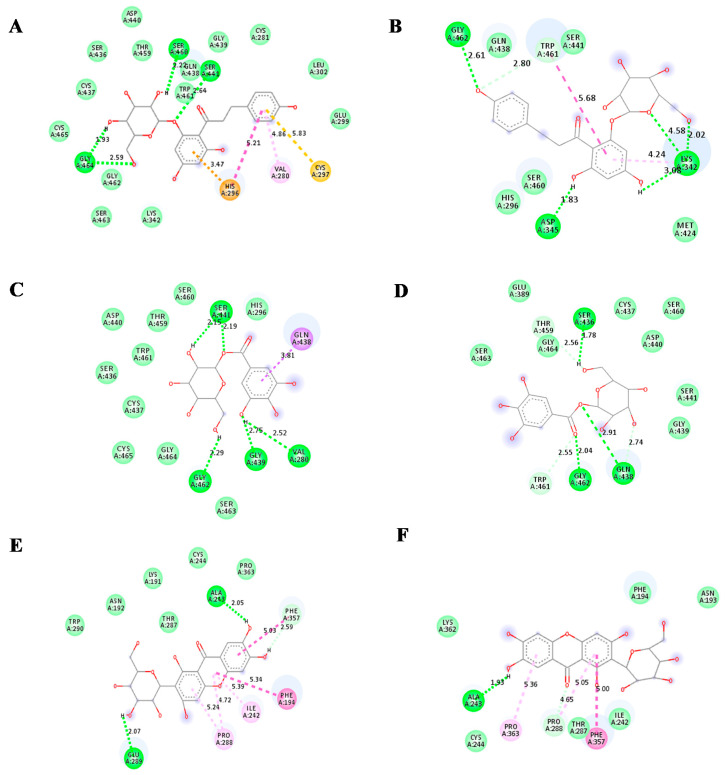
(**A**) Docked phlorizin-TMPRSS2 complex (t = 0), (**B**) phlorizin-TMPRSS2 complex after MD (t = 10ns), (**C**) Docked glucogallin-TMPRSS2 complex (t = 0), (**D**) glucogallin-TMPRSS2 complex after MD (t = 10ns), (**E**) Docked mangiferin-TMPRSS2 complex (t = 0) and (**F**) mangiferin-TMPRSS2 complex after MD (t = 10ns).

In the case of TMPRSS2, after MDS, the phlorizin-TMPRSS2 complex showed five new conventional bonds with LYS 342 (A) [three bonds], GLY 462 (A) [one bond], and ASP 345 (A) [one bond]. These five conventional bonds and bond length makes the RMSD stable (Figure 6A,B). In a second complex, i.e., glucogallin-TMPRSS2, after MDS three conventional hydrogen bonds have been formed with SER 436 (A), GLN 438 (A), and GLY 462 (A). The bond length of the GLY 462 (A) became shorter from 2.29 Å to 2.04 Å; this parameter makes the RMSD plots stable with less RMSD value (Figure 6C,D). Whereas in the third complex mangiferin-TMPRSS2 after MDS, only one conventional bond with ALA 243 (A) has been formed with less RMSD value (Figure 6E,F).

**Figure 7:**
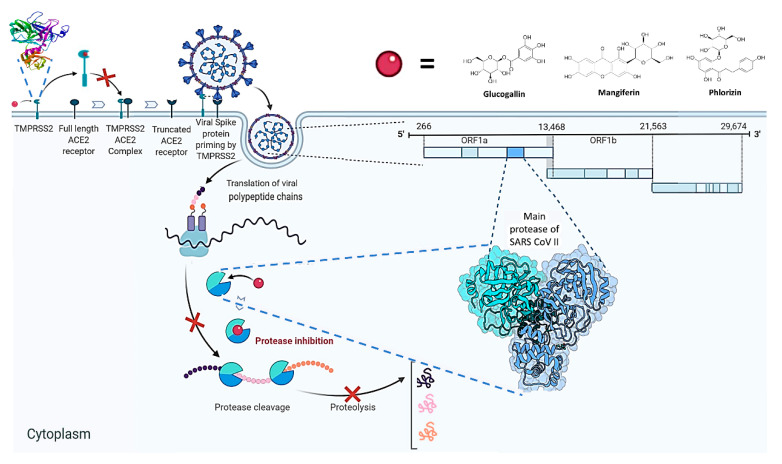
Hypothetical illustration of the anti-SARS-CoV-2 activity of the three polyphenolic compounds.

To further analyze the effect of these compounds on the active sites of the targets, the root means square fluctuations (RMSF) study has been performed. RMSF study of the three natural compounds with SARS-COV-2 Mpro showed very less fluctuations, and the value was found to be less than 0.2 nm, which indicates that the ligands bind properly with the active sites of the protein such as the reference compounds (Figure S3A). In the case of TMPRSS2, ligands-TMPRSS2 complexes showed little fluctuation upto amino acid 1800, but after that, it became stable. The RMSF values of all the natural products are less than 0.5 nm (Figure 6B). Furthermore, we analyzed the solvent-accessible surface area (SASA), to screen hydrophilic and hydrophobic residues of the target–protein and docked ligand-protein complexes. The SASA plots of the natural compounds docked with Mpro and TMPRSS2 showed less value compared to the un-docked protein targets (Figure S4). The radius of gyration (Rg) plot, which indicates the compactness of protein structure, was also performed. The selected natural compounds showed the Rg value between 2.20 to 2.25 nm for the Mpro. In the case of TMPRSS2, the phlorizin showed the plateau up to 1 ns w.r.t. TMPRSS2 protein and then regained the plateau phase nearly at 5 ns. The Rg value was found to be around 2.15 ± 0.05 nm. The second compound glucogallin showed plateau up to 2.5 ns and regains the same phase after 7.5 ns till the end of MDS with a Rg value of 2.15 ± 0.05 nm. The mangiferin showed the plateau phase with TMPRSS2 undocked protein from 1 ns to 3 ns with the Rg value of 2.15 ± 0.05 nm (Figure 7).

MM-PBSA analysis has been carried out on all the three target-ligand complexes to analyze the affinity of the natural compounds towards the protein molecules. The binding free energy calculation was carried out for 10,000 ps based on the MDS trajectories. The energy calculations provide solvation energy, binding energy, electrostatic energy, van der Waals energy, and SASA energy after the MD simulation process. In case of Mpro-ligand complexes, Mpro-phlorizin complex showed the highest binding energy of –9.65 ± 3.331 kcal/mol followed by glucogallin-Mpro and mangiferin-Mpro complexes showing binding energy of −8.222 ± 2.52 and −4.50 ± 8.37 kcal/mol, respectively. Whereas in the case of TMPRSS2-ligand complexes, mangiferin-TMPRSS2 complex showed the highest binding energy of −3.04 ± 3.98 kcal/mol followed by TMPRSS2-phlorozin andglucogallin-TMPRSS2 complexes with binding energy of −1.575 ± 4.83 kcal/mol and −1.100 ± 4.67 kcal/mol, respectively. The resulted energies by MM-PBSA of the MD trajectories (after 10ns) showed that the natural compounds still bind at active pockets of the protein. The energies of target-ligand complexes are listed in Table 4.

## 3. Discussion

Polyphenolic compounds are one of the groups of phenolic compounds that are already popular for their multiple medicinal activities. In search for some potential polyphenolic compounds against SARS-CoV-2, some in-silico studies have already showed that polyphenols from different natural sources such as black tea, green tea, mint, velvet bean, etc., were found to have inhibitory potentiality, and some flavonoid compounds were successfully repurposed for the management of the COVID-19 [27,28,29].Our current study is focused to screen three selected polyphenolic compounds present in fruits such as amla, apple, and mango as a potential inhibitor for SARS-CoV-2. In summarized form, we did the molecular docking study of the three selected natural polyphenolic compounds against the main protease of SARS-CoV-2 and TMPRSS2 protease. All the three natural compounds showed good affinity towards the viral main protease and TMPRSS2. As there is no 3D structure available for TMPRSS2, we have built a homology model using its protein sequence and 3D structure of Hepsin (TMPRSS1) on SWISS MODEL server. Analyzing the three ligand-TMPRSS2 complexes, we observed that the compounds phlorizin and glucogallin interacts with the residues of the homology model that are common with the primary sequence of Hepsin. This indicates the reliability of our homology model.However, interacting residues of mangiferin does not match with any common residues of the homologous sequence of Hepsin. Phlorizin showed the best affinity towards TMPRSS2, compared to mangiferin and glucogallin. Mangiferin showed the highest binding affinity of −8.5 Kcal/mol with SARS-CoV-2 main protease. Mangiferin was previously reported as an immune and respiratory stimulant agent along with anti-viral properties [13,14,15,16]. The rest of the two compounds also showed good binding affinity to the main protease. Analyzing the molecular docking result of three compounds in comparison to the reference compounds, we observed that they possess some common interacting residues. This indicates that the compounds are binding in the same pocket of the target proteins as the reference compounds. Glucogallin comes under the class of gallotannins, which have been reported to possess anti-viral property against Herpes simplex virus type 1 [18]. To analyze the pharmacokinetics and toxicity parameter, we used the pkCSM database. The properties of these compounds show that they can transport through specific membranes, distribute in the system, and bind to the specific proteins. The absorption of the molecules mainly depends upon the lipophilicity and hydrophilicity factors. All compounds have sugar moieties, which increase the water solubility. These compounds do not have any inhibition effect on liver enzymes (such as CYP1A2 etc.) that are involved in the metabolism of the drugs. Thus, the metabolism of anticancer, anticonvulsant, and antimalarial drugs remain unaffected. No interference with CYP2C19 and CYP2D9 enzymes involved in the metabolism of cardiovascular drugs and NSAIDs, respectively, was also noted. These compounds are not involved in drug-drug interaction. Whereas the server predicted that the reference compounds may have the inhibition activity on the liver enzymes that are involved in drug metabolism. The toxicity study of the polyphenolic compounds predicts that it is safe for animal and human consumption, but the reference compounds may have hepatotoxicity. The hepatoxicity of remdesivir have already been reported [30]. The molecular dynamic simulation showed that the selected natural compounds bind and stabilized the active site of both the proteins such as Mpro and TMPRSS2. The resultof the MM-PBSA free energy calculations suggested that the phlorizin have the lowest binding free energy towards the Mpro, followed by the glucogallin and mangiferin. For TMPRSS2, mangiferin showed the lowest binding free energy, followed by phlorizin and glucogallin.

## 4. Material and Methods

### 4.1. Preparation of Ligand and Protein

For this study, we have selected three natural compounds from different natural sources that were previously reported to have antiviral activity. To study the ligand-protein interaction, we have downloaded the “sdf” file of ligands from the PubChem. The protein crystalline structure of COVID-19 main protease (Mpro) with ligand (PDB-6LU7) was downloaded from the RCSB protein databank (www.rcsb.org). The protein structure of proteases was cleaned by removing the ligand, water molecule or hetatom by using biovia discovery studio and saved the protein in Protein Data Bank (PDB) format.

### 4.2. Homology Modelling

Homology modeling is extensively used to generate a valid structure of protein utilizing the amino acid sequence [31]. To date, the crystal structure of the TMPRSS2 enzyme was not established. We used the SWISS-MODEL server for the generation of the homology model of the TMPRSS2.The protein sequence of TMPRSS2 was given in the SWISS-MODEL server, the Human Hepsin TMPRSS1 (5ce1.1.A).1A shows a higher similarity of 33.82%. After preparing the 3D structure of the protein by SWISS-MODEL server, we downloaded the PDB file, and it was used for the molecular docking [32,33].

### 4.3. Procedure for Molecular Docking of Ligands and Protein

The molecular docking was performed by using PyRx version 0.8, together with AutoDock Vina. Both the protein and ligands were loaded to the PyRx virtual screening software. (https://pyrx.sourceforge.io/) [34]. The protease protein was put as fixed, but the ligands had rotatable torsions. The size of the box was designed around the center of protease protein, with an exhaustiveness parameter of 8 for all docking. The best binding affinity ligands were selected for the analysis of the inter-residue interaction. To cross check the docking scores of the compounds obtained from PyRx AutoDock Vina, we also used DockThor server [26]. The ligand and protein interaction were generated by the discovery studio visualize (DSV) version 2016, from BIOVIA.

### 4.4. ADME Prediction Study

Absorption, distribution, metabolism, and excretion (ADME) studies are important for better understanding of pharmacodynamic parameter of the selected molecules or drugs. For the analysis of the ADME parameter, the pkCSM database [35] was used. The user can select smiley string or smile file of the compounds. The database also provides the additional information about molecular properties (log P, rotatable bonds, acceptor, donors, and surface area), absorption (solubility, intestinal absorption, skin permeability, p-glycoprotein binding), distribution (volume distribution, protein binding, CNS, and blood–brain barrier permeability), metabolism (CYP substrate and inhibitors), and excretion (total clearance and renal transport).

### 4.5. Prediction of Target

This study has the importance in the identification of the possible targets, phenotypical side effects, or potential cross-reactivity of selected molecules [36]. The Swiss Target Prediction website has been used for the analysis of the possible target.

### 4.6. Prediction of Toxicity

The toxicity study is required for the analysis or prediction of the small molecules that are safe for human and animal uses. pkCSM online database has been used for the toxicity prediction. The molecules can be submitted in the form of smiles in the software. The database provides the AMES toxicity, human maximum tolerated dose, hERGI and II, LD50, hepato-toxicity, skin toxicity, T. pyriformis, and minnow toxicity of compounds [35].

### 4.7. Molecular Simulation and Energy Calculation

Molecular dynamics (MD) simulation was executed with the docked structure that have the minimum energy using GROMACS (Version 5.1.2) [37] with CHARMM36-march2019 [38] force field using the TIP3P model [39]. For the preparation of ligands, the CHARMM General Force Field server (https://cgenff.umaryland.edu/) was used. PBC was applied by generating a dodecahedron box. The system was neutralized by adding an adequate number of Na^+^ or Cl^−^ ions. Energy minimization was followed by system equilibration for 100ps at 300K using isochoric-isothermal (NVT) equilibration keeping the 2 fs time step. The isothermal-isobaric (NPT) ensemble was performed for 100 ps at 300 K with 2 fs time step. Electrostatic and van der Waals interactions cut-offs for both NVT and NPT were kept at 1.2nm. For calculation of long-range interactions, the smooth particle mesh Ewald (PME) method was applied. Further, 10,000 ps MD-simulation was performed using the same cut-off. Further 10ns trajectories were submitted to MM-PBSA for energy calculation with 10,000 frames [39,40].

## 5. Conclusions

From our data, we can conclude that the natural polyphenolic compounds, i.e., glucogallin, mangiferin, and phlorizin, may have the potential to be used as safe protease inhibitors, which may act by inhibiting TMPRSS2 and SARS-CoV-2 main protease, thus preventing SARS-CoV-2 spike protein priming and viral protein post-translational modification (Figure 7). Other advantages of these compounds are that they possess good absorption, good solubility in water, no inhibitory effect on liver enzymes, no effect on the metabolism of essential drugs such as anticancer, anticonvulsant, and anti-coagulant drugs. The potentiality of these compounds can be further verified and may be confirmed by in vitro study.

## Figures and Tables

**Table 1:** Predicted interactive residues of SARS-CoV-2 main protease with ligands.

Compound	Number of Conventional H Bonding	Residue Receptor	Bond Length(Å)	Docking Score (kcal/mol)	Actual Residue by Experimental Crystal Structure	Ref.
Remdesivir	5	THR199(A), ASP289(A), LYS137(A), ARG 131(A), LEU287(A)	2.51, 2.66, 2.91 2.75, 3.10	−7.9	-	-
N3	8	HIS41(A), THR190(A), GLU166(A), GLN189(A), THR26(A), GLY143(A), CYS145(A) (2H bonding)	2.65, 2.42, 2.65, 1.81, 3.10, 1.98, 3.40, 3.45	−7.5	GLN 189 (A),THR190(A),GLU166(A),PHE140(A),HIS164(A) and GLY 143(A)	[24]
X77	5	HIS164(A), GLY143(A), ASN142(A), GLU166(A), PHE140(A)	2.35, 3.09, 2.05, 2.19, 2.73	−8.6	GLY143(A), GLU166(A), ASN142(A) and CYS145(A)	[25]
Glucogallin	6	CYS145(A), SER144(A), GLY143(A) (2H bonding), HIS163(A), MET165(A)	3.3, 2.37, 2.40, 1.97, 2.40, 3.05	−7.0	-	-
Mangiferin	6	LEU141(A), SER144(A), ASN142(A) (2H bonding),THR 190(A) (2H bonding)	2.26,2.80, 2.72 2.12, 2.46,1.91	−8.5	-	-
Phlorizin	6	GLN189(A), MET49(A), CYS145(A), MET165(A), GLY143(A), SER144(A)	2.25, 2.98,3.75,2.88,2.59, 2.73	−7.9	-	-

**Table 2:** Predicted interactive residues of TMPRSS2 with ligands.

Compound	Number of H Bonding	Residue Receptor	Bond Length (Å)	Docking Score (kcal/mol)
Camostat mesylate	4	GLY464(A) (2H bonding), CYS465(A), HIS296(A), ASP 435(A)	2.30, 2.37, 2.92, 2.58,2.14	−7.1
Glucogallin	4	SER441(A) (2H bonding), VAL280(A), GLY439(A), GLY 462(A)	2.15, 2.19, 2.52, 2.75, 2.29	−6.9
Mangiferin	2	ALA 243(A), GLU 289(A)	2.05, 2.07	−6.9
Phlorizin	4	SER460(A), SER441(A), GLY 464(A) (2H bonding)	2.20, 2.64, 1.97, 2.59	−7.7

**Table 3:** Prediction of toxicity study of the ligands.

Compound	Maximum Tolerated Dose(Human) (log mg/kg/day)	Oral Rat Acute Toxicity (LD50) (mol/kg)	Oral Rat Chronic Toxicity (LOAEL) (log mg/kg bw/day)	T.PyriformisToxicity (log ug/L)	Minnow Toxicity (log mM)	Ames Toxicity	Hepato-Toxicity	Skin Sensitivity	hERGI/II
Remdesivir	0.15	2.043	1.639	0.285	0.291	NO	YES	NO	NO/YES
X77	0.601	2.396	1.528	0.285	2.563	YES	YES	NO	NO/YES
N3	0.305	2.344	3.084	0.287	−2.205	YES	YES	NO	NO/YES
Camostat mesylate	0.133	2.319	2.81	0.285	0.524	NO	NO	NO	NO
Mangiferin	0.58	2.396	4.277	0.285	5.898	NO	NO	NO	NO
Glucogallin	0.238	2.314	3.112	0.285	6.151	NO	NO	NO	NO
Phlorizin	0.555	2.494	4.667	0.285	6.334	NO	NO	NO	NO

**Table 4:** Predicted MM-PBSA analysis for calculation of thermodynamics parameters.

Target-Ligand Complex	Binding Energy (kcal/mol)	Solvation Energy (kcal/mol)	Electrostatic Energy (kcal/mol)	Van der WaalsEnergy (kcal/mol)	SASA Energy (kcal/mol)
Mpro-Remdesivir	−5.4127 ± 4.24	36.394 ± 8.92	−12.87 ± 4.90	−25.58 ± 5.40	−3.342 ± 0.53
Mpro-X77	−14.16 ± 11.478	35.77 ± 20.88	−9.35 ± 5.597	−36.71 ± 19.14	−3.879 ± 2.05
Mpro-N3	−16.58 ± 8.263	57.78 ± 26.110	−18.49 ± 8.145	−50.04 ± 20.54	−5.834 ± 2.39
CamostatMesylate-TMPRSS2	−34.6558 ± 4.34	−76.79 ± 16.5	−53.95 ± 7.26	−34.70 ± 4.34	−4.349 ± 0.35
Mpro-phlorizin	−9.65 ± 3.33	36.80 ± 6.52	−10.1 ± 3.40	−32.29 ± 5.15	−4.02 ± 0.45
Mpro-Glucogallin	−8.22 ± 2.52	34.190 ± 2.78	−10.34 ± 6.28	−29.56 ± 2.43	−3.55 ± 0.25
Mpro-Mangiferin	−4.50 ± 8.37	33.33 ± 23.71	−6.419 ± 5.76	−24.33 ± 12.7	−3.15 ± 1.64
TMPRSS2-Phlorizin	−1.57 ± 4.83	41.08 ± 9.90	−11.67 ± 5.84	−27.39 ± 5.29	−3.59 ± 0.45
TMPRSS2-Glucogallin	−1.10 ± 4.67	40.68 ± 7.25	−15.63 ± 4.89	−22.96 ± 3.52	−3.18 ± 0.26
TMPRSS2-Mangiferin	−3.04 ± 3.98	46.366 ± 10.41	−19.34 ± 8.453	−26.40 ± 3.01	−3.65 ± 0.25

## Supplementary Materials

The following are available online, Figure S1: Ramachandran plot of SWISS-Model-predicted structure of TMPRSS2, 92.7% favored region. 6.7% allowed region and 0.6% outlier region.Figure S2: RMSD plot of protein and protein-ligand complexes, (A) Mpro and (B) TMPRSS2. Figure S3: RMSF plot of protein and protein-ligand complexes, (A) Mpro and (B) TMPRSS2. Figure S4: SASA plot of protein and protein-ligand complexes, (A) Mpro and (B) TMPRSS2. Figure S5: Rg plot of protein and protein-ligand complexes, (A) Mpro and (B) TMPRSS2. Table S1: Predicted molecular properties of the Compounds.Table S2: Predicted Absorption parameter of the compounds. Table S3: Predicted Distribution and Excretion parameter of the compounds. Table S4: Predicted metabolism parameter of the compounds. Table S5: Predicted docking score of ligands with TMPRSS2 by PyRxVina and DockThor software. Table S6: Predicted docking score of ligands with Mpro by PyRxVina and DockThor software.

## Abbreviations

CoV	Coronavirus
COVID-19	Coronavirus disease-19
GROMACS	Groningen Machine for Chemical Simulation
hERG	Human Ether-a-go-go-Related Gene
HSV-1	Herpes Simplex Virus-1
log P	Partition Co-efficient
MD	Molecular Dynamics
Mpro	main protease
RMSD	root mean square division
RMSF	root mean square fluctuation
RCSB	Research Collaboratory for Structural Bioinformatics
SARS-CoV-2	Severe Acute Respiratory Syndrome-Coronavirus-2
