# Supplementary material for: Protease Inhibitory Effect of Natural Polyphenolic Compounds on SARS-CoV-2: An In Silico Study
Source: Molecules. 2020 Oct 10;25(20):4604. doi: 10.3390/molecules25204604 (PMC7587198; doi:10.3390/molecules25204604)
Supplement: Supplementary file 1 [file molecules-25-04604-s001.pdf]

Supplementary Materials for

Protease Inhibitory Effect of Natural Polyphenolic Compounds on SARS-CoV-2: An In-Silico Study

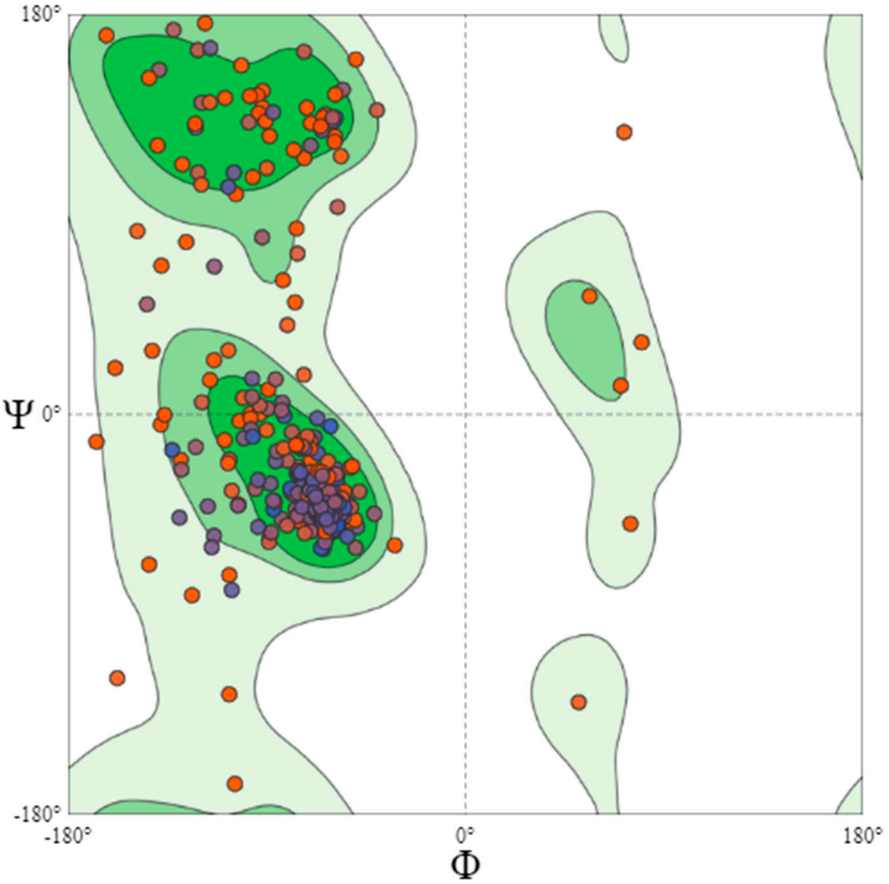

Figure S1. Ramachandran plot of SWISS-Model-predicted structure of TMPRSS2, 92.7% favored region. 6.7% allowed region and 0.6% outlier region.

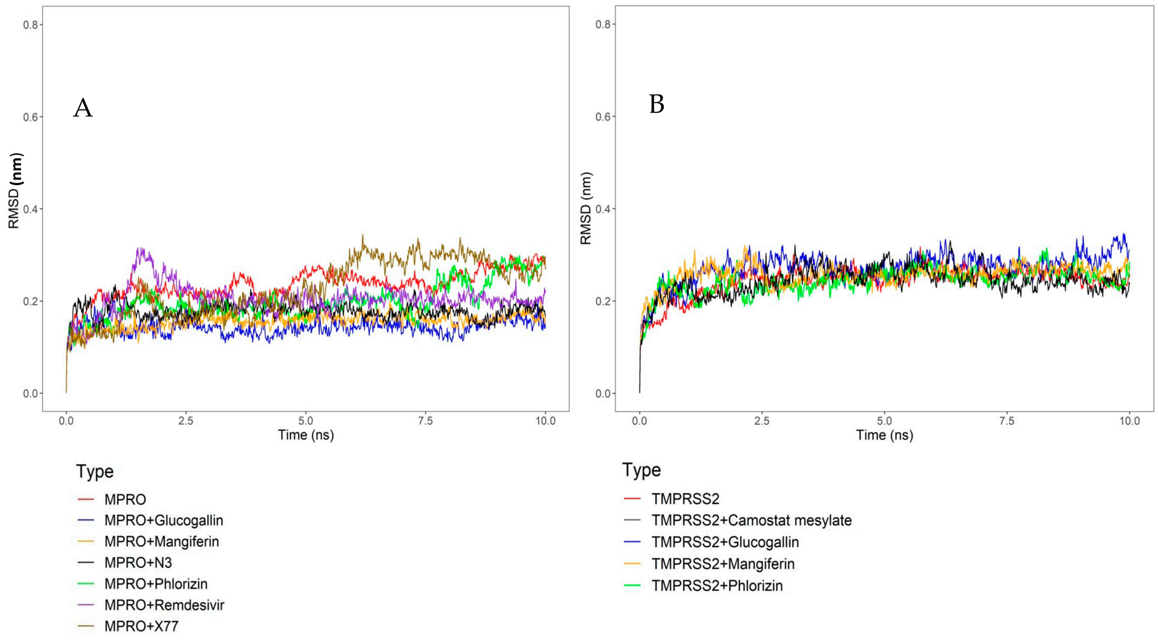

Figure S2. RMSD plot of protein and protein-ligand complexes, (A) Mpro and (B) TMPRSS2.

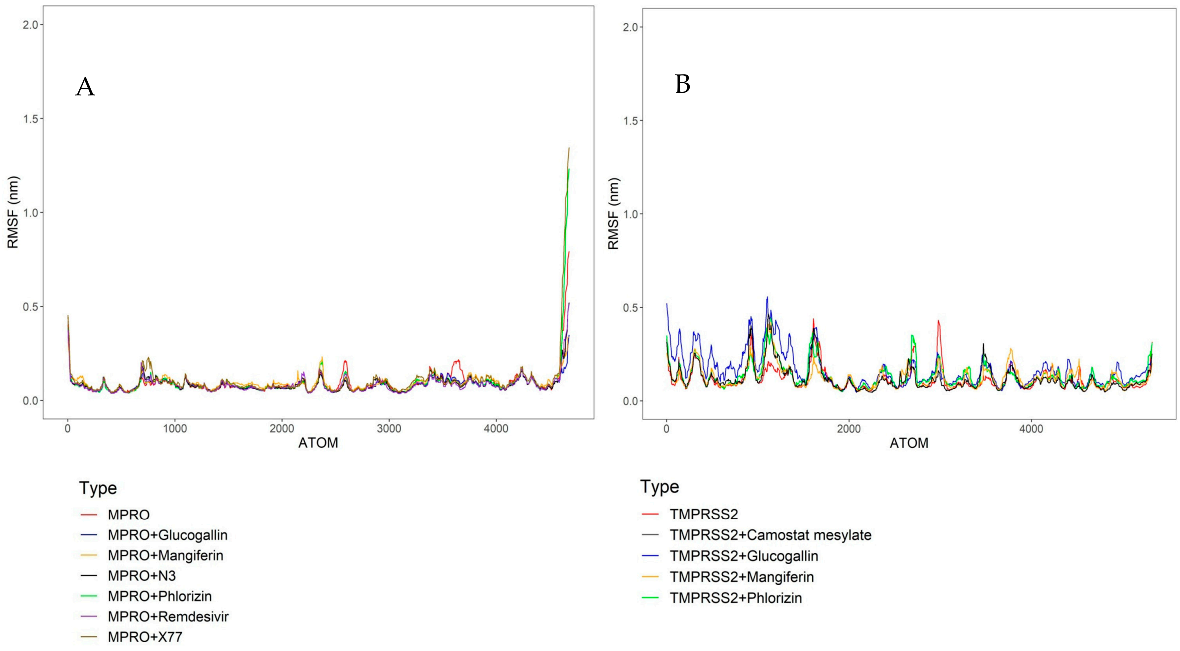

Figure S3. RMSF plot of protein and protein-ligand complexes, (A) Mpro and (B) TMPRSS2.

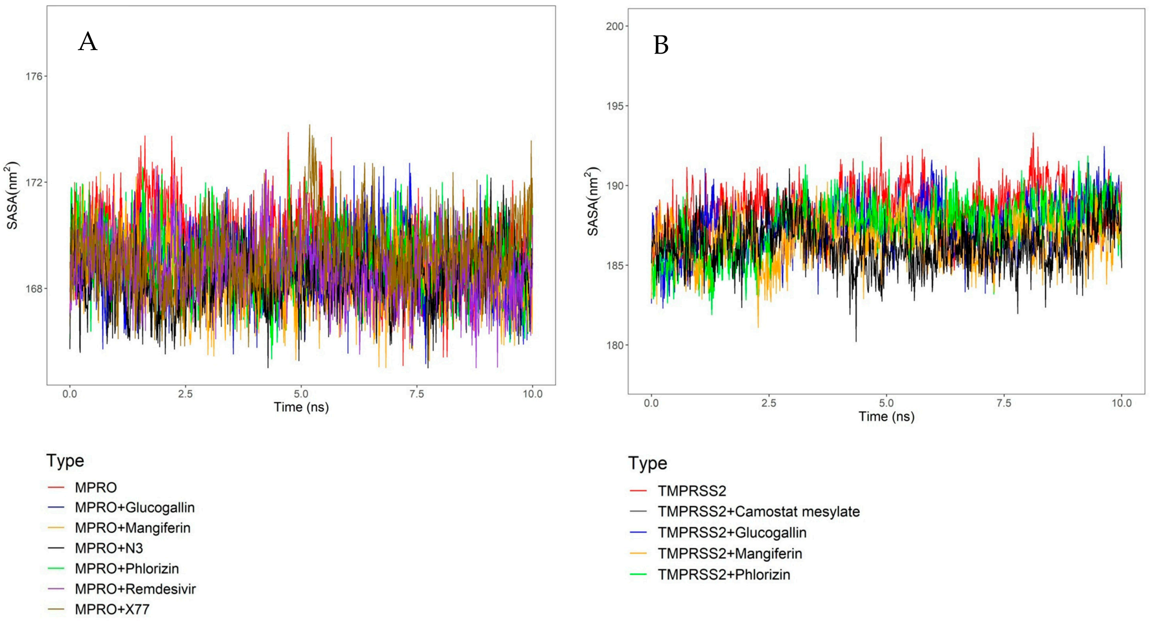

Figure S4. SASA plot of protein and protein-ligand complexes, (A) Mpro and (B) TMPRSS2.

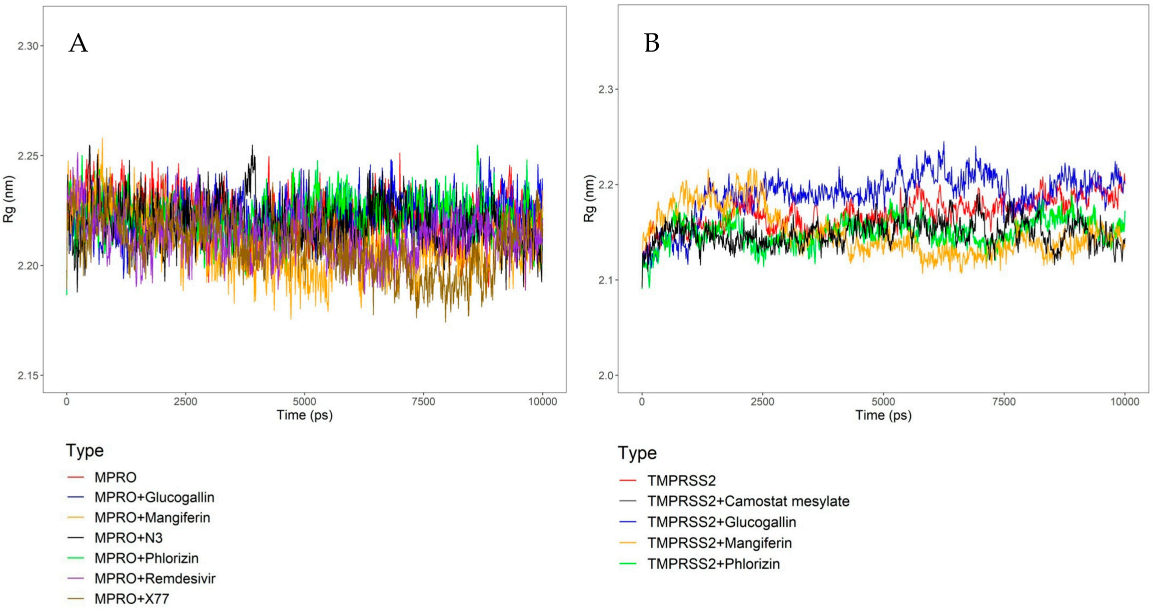

Figure S5. Rg plot of protein and protein-ligand complexes, (A) Mpro and (B) TMPRSS2.

Table S1. Predicted molecular properties of the Compounds.

Compound	Molecular Weight(g/mol)	LogP	Rotatable Bonds	Acceptors	Donors	Surface Area(Å^2)
Remdesivir	602.585	2.31218	13	13	4	242.488
X77	459.594	4.9392	6	4	2	200.565
N3	426.516	5.9925	10	5	3	187.819
Camostat mesylate	494.526	0.4886	7	8	3	196.454
Glucogallin	332.261	-1.6283	3	10	6	129.248
Mangiferin	422.342	-0.7165	2	11	8	166.412
Phlorizin	436.413	-0.2024	7	10	7	176.715

Table S2. Predicted Absorption parameter of the compounds.

Compound	Water solubility(log mol/L)	Caco2 permeability (log Papp in 10 $^{-6}$cm/s)	Intestinal absorption (human) (% Absorbed)	Skin Permeability(log Kp)	P-glycoprotein substrate	P-glycoprotein I inhibitor	P-glycoprotein II inhibitor
Remdesivir	-3.07	0.635	71.109	-2.735	Yes	Yes	No
X77	-2.878	1.29	89.686	-2.735	Yes	Yes	Yes
N3	-3.72	0.519	86.997	-2.735	Yes	Yes	Yes
Camostat mesylate	-2.946	-0.552	20.833	-2.735	Yes	No	No
Glucogallin	-2.626	-0.884	31.251	-2.735	Yes	No	No
Mangiferin	-2.918	-0.926	46.135	-2.735	Yes	No	No
Phlorizin	-2.493	0.189	37.825	-2.735	Yes	No	No

Table S3. Predicted Distribution and Excretion parameter of the compounds.

Compound	VDss (human) (log L/kg)	Fraction unbound (human) (Fu)	BBB permeability (log BB)	CNS permeability (log PS)	Total Clearance (log ml/min/kg)	Renal OCT2 substrate
Remdesivir	0.307	0.005	-2.056	-4.675	0.198	No
X77	0.656	0.262	-0.899	-2.31	0.671	Yes
N3	-0.906	0.015	-0.173	-1.989	0.08	No
Camostat mesylate	-0.37	0.183	-1.398	-4	0.374	No
Glucogallin	1.332	0.58	-1.514	-3.729	0.584	No
Mangiferin	1.364	0.289	-1.573	-4.211	0.347	No
Phlorizin	0.667	0.27	-1.133	-3.983	0.141	No

Table S4. Predicted metabolism parameter of the compounds.

Compound	CYP2D6 substrate	CYP3A4 substrate	CYP1A2 inhibitior	CYP2C19 inhibitior	CYP2C9 inhibitior	CYP2D6 inhibitior	CYP3A4 inhibitior
Remdesivir	No	Yes	No	No	No	No	No
X77	No	No	No	No	No	No	YES
N3	YES	YES	YES	YES	YES	No	YES
Camostat mesylate	No	YES	No	No	No	No	YES
Glucogallin	No	No	No	No	No	No	No
Mangiferin	No	No	No	No	No	No	No
Phlorizin	No	No	No	No	No	No	No

Table S5. Predicted docking score of ligands with TMPRSS2 by PyRx Vina and DockThor server.

Compound	Docking Score With Pyrx Vina (kcal/mol) for TMPRSS2	Docking Score With Dock Thor (kcal/mol) TMPRSS2
Glucogallin	-6.9	-6.4
Mangiferin	-7.0	-7.3
Phlorizin	-7.7	-7.5
Camostat mesylate	-7.1	-7.1

Table S6. Predicted docking score of ligands with Mpro by PyRx Vina and DockThor server.

Compound	Docking Score With Pyrx Vina (kcal/mol) for Mpro	Docking Score With Dock Thor (kcal/mol) Mpro
Glucogallin	-7.0	-7.5
Mangiferin	-8.5	-8.0
Phlorizin	-7.9	-7.2
N3	-7.5	-7.5
Remidesivir	-7.9	-8.3
X77	-8.5	-7.8
